# The EMA Assessment of Asciminib for the Treatment of Adult Patients With Philadelphia Chromosome-Positive Chronic Myeloid Leukemia in Chronic Phase Who Were Previously Treated With at Least Two Tyrosine Kinase Inhibitors

**DOI:** 10.1093/oncolo/oyad119

**Published:** 2023-05-04

**Authors:** C Mircea S Tesileanu, Sotirios Michaleas, Rocio Gonzalo Ruiz, Segundo Mariz, Babs O Fabriek, Paula B van Hennik, Jutta Dedorath, Bruna Dekic, Christoph Unkrig, Andreas Brandt, Janet Koenig, Harald Enzmann, Julio Delgado, Francesco Pignatti

**Affiliations:** Oncology and Hematology Office, European Medicines Agency, Amsterdam, The Netherlands; Department of Neurology, The Brain Tumor Center, Erasmus MC Cancer Institute, Rotterdam, The Netherlands; Oncology and Hematology Office, European Medicines Agency, Amsterdam, The Netherlands; Oncology and Hematology Office, European Medicines Agency, Amsterdam, The Netherlands; Orphan Medicines Office, European Medicines Agency, Amsterdam, The Netherlands; Medicines Evaluation Board, Utrecht, The Netherlands; Medicines Evaluation Board, Utrecht, The Netherlands; Committee for Medicinal Products for Human Use (CHMP), European Medicines Agency, Amsterdam, The Netherlands; Federal Institute for Drugs and Medical Devices, Bonn, Germany; Federal Institute for Drugs and Medical Devices, Bonn, Germany; Federal Institute for Drugs and Medical Devices, Bonn, Germany; Federal Institute for Drugs and Medical Devices, Bonn, Germany; Committee for Medicinal Products for Human Use (CHMP), European Medicines Agency, Amsterdam, The Netherlands; Federal Institute for Drugs and Medical Devices, Bonn, Germany; Committee for Medicinal Products for Human Use (CHMP), European Medicines Agency, Amsterdam, The Netherlands; Federal Institute for Drugs and Medical Devices, Bonn, Germany; Oncology and Hematology Office, European Medicines Agency, Amsterdam, The Netherlands; Department of Hematology, Hospital Clinic, Barcelona, Spain; Oncology and Hematology Office, European Medicines Agency, Amsterdam, The Netherlands

**Keywords:** asciminib, chronic myeloid leukemia, EMA, tyrosine kinase inhibitor, BCR-ABL1

## Abstract

Asciminib is an allosteric high-affinity tyrosine kinase inhibitor (TKI) of the BCR-ABL1 protein kinase. This kinase is translated from the Philadelphia chromosome in chronic myeloid leukemia (CML). Marketing authorization for asciminib was granted on August 25, 2022 by the European Commission. The approved indication was for patients with Philadelphia chromosome-positive CML in the chronic phase which have previously been treated with at least 2 TKIs. Clinical efficacy and safety of asciminib were evaluated in the open-label, randomized, phase III ASCEMBL study. The primary endpoint of this trial was major molecular response (MMR) rate at 24 weeks. A significant difference in MRR rate was shown between the asciminib treated population and the bosutinib control group (25.5% vs. 13.2%, respectively, *P* = .029). In the asciminib cohort, adverse reactions of at least grade 3 with an incidence ≥ 5% were thrombocytopenia, neutropenia, increased pancreatic enzymes, hypertension, and anemia. The aim of this article is to summarize the scientific review of the application which led to the positive opinion by the European Medicines Agency’s Committee for Medicinal Products for Human Use.

## Teaser

This article summarizes the scientific review of the application that led to the positive opinion of asciminib by the European Medicines Agency's Committee for Medicinal Products for Human Use.

## Introduction

Chronic myeloid leukemia (CML) is a rare hematological malignancy of which the Philadelphia chromosome is the pathological hallmark.^1,2^ On this chromosome, a reciprocal translocation between chromosomes 9 and 22 has occurred. This translocation results in a fusion gene of BCR activator of RhoGEF and GTPase (*BCR*) and ABL proto-oncogene 1, non-receptor tyrosine kinase (*ABL1*).^3,4^ The BCR-ABL1 protein translated from the fusion gene is a constitutively active protein kinase which activates the cell cycle and inhibits DNA repair.^5-7^ The BCR-ABL1 protein can be specifically targeted with tyrosine kinase inhibitors (TKIs). Imatinib was the first TKI to be developed to target the BCR-ABL1 protein.^8-11^ TKIs are particularly effective when CML is treated in the chronic phase (CML-CP). The prognosis for treated CML-CP patients can approach the life expectancy of the general ­population.^1,12-14^ An unmet medical need remains for CML-CP patients with TKI-resistant tumors, or CML-CP patients who had to discontinue TKI treatment due to toxicity. Asciminib, branded as Scemblix, provides an alternative for standard TKI treatment by utilizing a novel mechanism of action.^15^

In this review, we summarize the assessment by the European Medicines Agency (EMA) of an initial request for “orphan drug” designation and marketing authorization for asciminib in patients with Philadelphia ­chromosome-positive CML-CP which have previously been treated with at least 2 TKIs.^16-18^ Asciminib has received an initial “orphan drug” designation after a positive decision by the Committee for Orphan Medicinal Products (COMP) was ratified by the European Commission (EC) on March 24, 2020. This designation was maintained in the Community Register of Orphan Medicinal Products after reevaluation by the COMP on July 14, 2022 as highlighted in Box 1.^19^ The marketing authorization application was submitted by Novartis Europharm Limited (Dublin, Ireland) on June 22, 2021. Marketing authorization was granted on August 25, 2022 by the EC.^20^

## Standard-of-Care, Nonclinical Aspects, and Clinical Pharmacology

Current first-line treatment of CML-CP consists of TKIs such as imatinib, nilotinib, dasatinib, and bosutinib.^21-24^ These TKIs competitively bind to the ATP-binding site on the BCR-ABL1 active site and hereby inhibit the protein kinase activity.^25^ In case of either resistance of the CML-CP to the initial TKI, or intolerance of the patient to the initial TKI treatment a switch can be made to one of the other TKIs.^14^ If CML-CP TKI-resistance is caused by the *BCR-ABL*^*T315I*^ mutation, a switch should be made specifically to the TKI ponatinib because all other previously mentioned TKIs would then be ineffective.^26^ However, cardiovascular toxicity is a well-recognized complication of ponatinib treatment.^27^

In contrast to other TKIs, asciminib allosterically binds to the myristoyl pocket of the BCR-ABL1 protein, locking it into an inactive conformation without blocking the kinase ATP-binding site.^15,28,29^ This unique mechanism of action allows for targeting of both native and mutated BCR-ABL1 proteins, including those with the *BCR-ABL*^*T315I*^ mutation.^29^ Furthermore, preclinical data showed that asciminib inhibits ABL1 tyrosine kinase in vitro, inhibits cell growth and proliferation in BCR-ABL1 expressing cells, and inhibits tumor growth in mouse xenograft BCR-ABL1 expressing CML.^30^

## Pivotal Clinical Trial

The pivotal trial assessed by the EMA was the open-label, randomized, phase III ASCEMBL study in which patients with Philadelphia chromosome-positive CML-CP (*n* = 233) previously treated with at least 2 TKIs were treated with either asciminib 40 mg twice daily (*n* = 157) or bosutinib 500 mg once daily as a control therapy (*n* = 76).^31^ Patients with T315I and/or V299L mutations at any time prior to study entry were excluded from the ASCEMBL trial because of the known resistance to bosutinib.^32^ Bone marrow and peripheral blood samples were extracted at 24 and 96 weeks after treatment and assessed for complete cytogenetic response (CCyR) and major molecular response (MMR). CCyR was defined as an absence of Philadelphia-positive metaphases in bone marrow with a minimum of 20 metaphases examined, and MMR was defined as quantitative reverse-transcriptase-polymerase-chain-reaction transcript levels in blood samples of BCR-ABL1 ≤ 0.1% on the international scale. The primary endpoint of the trial was MMR rate at 24 weeks, and the major secondary endpoints were MMR rate at 96 weeks and CCyR rate at 24 and 96 weeks. For the primary endpoint, the difference in MMR rate at 24 weeks was significant with an MMR rate of 25.5% for the asciminib-treated population against 13.2% for the bosutinib control group (*P* = .029). At 96 weeks, this difference increased to 37.6% in the asciminib cohort compared to 14.5% in the bosutinib cohort (*P* = .001). CCyR rates at 24 and 96 weeks favored the asciminib cohort as well with a response rate difference of 16.6% [95% confidence interval (CI), 2.3-30.9], and 23.9% [95% CI, 10.3-37.4], respectively. In the asciminib cohort, adverse reactions of at least grade 3 with an incidence  ≥ 5% were thrombocytopenia, neutropenia, increased pancreatic enzymes, hypertension, and anemia. In the same population, serious adverse reactions with an incidence  ≥ 1% were pleural effusion, lower respiratory tract infections, thrombocytopenia, pyrexia, pancreatitis, non-cardiac chest pain, and vomiting. The most important favorable and unfavorable effects of treatment with asciminib are summarized in Table 1.

**Table 1.** The effects table of asciminib for the treatment of adult patients with Philadelphia chromosome-positive CML-CP previously treated with 2 or more TKIs.

Effect	Asciminib	Bosutinib	Reference
Responses, in % [95%CI, %-%]			
MMR rate at 24 weeks	25.5 [18.9-33.0]	13.2 [6.5-22.9]	^1^
MMR rate at 96 weeks	37.6 [30.0-45.7]	15.8 [8.4-26.0]	^1^
Duration of MMR at 24 weeks	95.4 [82.8-98.8]	100 [NE-NE]	^1^
Duration of MMR at 72 weeks	96.7 [87.4-99.2]	92.9 [59.1-99.0]	^1^
CCyR rate at 24 weeks	40.8 [31.2-50.9]	24.2 [14.2-36.7]	^1^
CCyR rate at 96 weeks	39.8 [30.3-49.9]	16.1 [8.0-27.7]	^1^
Adverse events, incidence of all grades/grade ≥3 in %			
Thrombocytopenia	23.1/17.9	14.5/6.6	^1^
	27.8/11.1		^2^
	27.5/18.5		^3^
Neutropenia	19.2/15.4	17.1/11.8	^1^
	22.2/11.1		^2^
	19.4/15.7		^3^
Anemia	9.6/1.3	7.9/3.9	^1^
	16.7/0		^2^
	12.9/5.3		^3^
Hypertension	12.2/5.8	5.3/3.9	^1^
	33.3/22.2		^2^
	18.5/8.7		^3^
Increased lipase	5.1/3.8	6.6/5.3	^1^
	22.2/16.7		^2^
	21.3/12.3		^3^
Pancreatitis	1.1/0.6		^3^

Reference groups are (1) the ASCEMBL trial; (2) the X2101 study, asciminib 80 mg once a day; and (3) pooled data of the ASCEMBL trial and the X2101 study, asciminib all dosages. Duration of MMR at 24 and 72 weeks was defined as the percentage of patients maintaining MMR for at least the respective number of weeks.

Abbreviations: MMR, major molecular response; CCyR, complete cytogenetic response; NE, not estimable.

## Benefit-Risk Assessment

As mentioned previously, superiority of asciminib over bosutinib in patients previously treated with 2 or more TKIs was demonstrated in the pivotal ASCEMBL study. The efficacy of asciminib as compared to bosutinib was demonstrated in patients who received 2, 3, or at least 4 prior TKI therapies (24 weeks MMR rate of asciminib vs. bosutinib: 2 prior TKIs 30.3% vs. 18.2%, 3 prior TKIs 22.6% vs. 12.1%, and 4 or more prior TKIs 6.7% vs. 0%). Using MMR rate as a surrogate endpoint for overall survival is well established in CML clinical trials and treatment guidelines.^14^ However, the selection of 24 weeks MMR as a primary endpoint for CML-CP did not comply with the EMA’s Anticancer guideline for superiority trials against a licensed comparator, as the guideline describes the need for longer follow-up data of at least 18 months.^33^ After a major objection on this topic in the first round of the procedure, the CHMP utilized the positive results of secondary endpoints, ie, MMR at 96 weeks and the percentage of patients maintaining MMR for at least 72 weeks, to further substantiate efficacy of asciminib at and beyond the required time point of 18 months MMR.

Bosutinib was agreed to be a valid comparative therapy, as it has been approved by the European Commission for the indication of CML-CP previously treated with at least 2 TKIs.^34^ The open-label design of the ASCEMBL study could not be avoided due to the selection of bosutinib as the comparative therapy, because asciminib is administered in a fasted state and bosutinib is administered with food. Blinding by double-dummy treatment was deemed to be too complex and at risk of creating administration and dosing errors. Likewise, an open-label design has been acceptable in previous CHMP’s assessments of TKIs for the treatment of CML-CP.

The asciminib cohort of the ASCEMBL study showed less adverse events than the bosutinib cohort resulting in a favorable relative safety profile. Myelosuppression was reported as a severe adverse event in the asciminib cohort, but it was reversible and managed by temporarily withholding treatment. Clinical safety results were supported by data from one arm of the independent, phase I open-label X2101 study which treated CML-CP patients with single-agent asciminib at different doses. Regarding clinical safety, the assessment of cardiovascular toxicity such as arterial occlusive events was highlighted, as it is a known toxicity of ponatinib treatment.^14,27^ Post hoc analyses of the pooled data from the ASCEMBL study, and the X2101 study showed no significant association between arterial occlusive events and daily asciminib dose, or duration of exposure. Arterial occlusive events occurred primarily in CML-CP patients with cardiovascular risk factors at baseline, a medical history of cardiovascular events, or prior treatment with cardiotoxic TKIs such as nilotinib or ponatinib. Pancreatic toxicity, myelosuppression, and QTc prolongation were considered important identified risks, while hepatotoxicity, and hepatitis B virus infection reactivation were considered important potential risks. Long-term safety and identified risks of asciminib treatment will be further assessed postauthorization in 4 ongoing safety studies.

## Marketing Authorization Outside of the EU

The United States Food and Drug Administration (FDA) approved marketing authorization for asciminib on October 29, 2021 for 2 indications. First, matching the approval at the EMA, asciminib was approved for the treatment of adult patients with Philadelphia chromosome-positive CML-CP previously treated with 2 or more TKIs. Second, asciminib was approved for the treatment of adult patients with Philadelphia chromosome-positive CML-CP with the T315I mutation. The FDA approval for the Philadelphia positive CML-CP population with the T315I mutation was based on the X2101 study. The daily dose of asciminib in the X2101 study for this subset of patients was 5 times higher than in the ASCEMBL trial. This indication, based on a phase I study and a higher asciminib dosage, was not requested by the company in the initial EMA marketing authorization application. As a result, the FDA approval included this second indication, but it was not included in the EMA approval.

## Conclusions

Based on the review of data on quality, safety, and efficacy, the EMA CHMP concluded by consensus that the risk‐benefit balance of asciminib was favorable for the treatment of adult patients with Philadelphia chromosome-positive CML-CP previously treated with 2 or more TKIs. The marketing authorization holder (MAH) is required to submit standard Periodic Safety Update Reports and an updated Risk Management Plan when necessary. Furthermore, the MAH is required to complete the 4 ongoing Post-Authorization Safety Studies.

**Box 1. “Orphan drug” Designation**

“Orphan drugs” are medicinal products intended for rare indications for which research and development are incentivized by the regulatory bodies to increase product profitability. The EMA defines “orphan drugs” accordant with 3 major criteria: (1) the prevalence of the “orphan condition” is less than 5 in 10 000 EU citizens; (2) the “orphan condition” is a life-threatening or chronically debilitating disease; and (3) the “orphan drug” must be of significant benefit in the treatment of the “orphan condition.” Regarding the latter criterium, significant benefit denotes drug-induced improvement of patient outcome as opposed to all other available treatment for the same “orphan condition,” and it also considers complete or partial overlap of the therapeutic indication for the “orphan drug” as compared to other products authorized for the same “orphan condition.” The main incentive for obtaining “orphan drug” designation at the EMA is 10 years of market exclusivity for the “orphan condition” after receiving market authorization. Other incentives offered by the EMA are (a) “orphan drug” tailored scientific advice known as protocol assistance, (b) central assessment of the “orphan drug” for the entire EU, (c) fee reductions on regulatory activities, (d) administrative and procedural assistance for micro-, small-, and ­medium-sized enterprises, and (e) an additional 2 years of market exclusivity for “orphan drugs” with an approved pediatric investigation plan. As an example for “orphan drug” designation assessment at the EMA, asciminib was assessed for the scope of the treatment of CML according to the 3 major criteria. First, the estimate of CML prevalence was noted to be 1.4 in 10,000 EU citizens, with a highest reported national prevalence of 2 in 10,000 Belgian citizens. Second, CML was considered both a life-threatening and chronically debilitating disease due to the presence and/or risk of bone marrow dysfunction, intracranial, and gastrointestinal hemorrhages, disseminated intravascular coagulation, and severe infections. Third, the evidence for significant benefit of asciminib over bosutinib was provided in the comparative trial the applicant submitted in their marketing authorization application. The significant benefit of asciminib over ponatinib, another TKI-resistant CML drug, was assessed with matching-adjusted indirect comparison (MAIC), as there was no direct comparison trial conducted by the sponsor. The MAIC analysis compared data of the ASCEMBL trial (asciminib) with 203 patients with CML-CP from the PACE (ponatinib) trial, and MAIC-adjusted MMR differences were non-significant at 24 and 48 weeks. Moreover, subgroup analysis of the ASCEMBL trial was indicative for efficacy of asciminib in patients previously treated with ponatinib. Therefore, significant benefit of asciminib was established in patients with CML-CP especially for those resistant to or intolerant of ponatinib. In conclusion, all the requirements for “orphan drug” designation were met for the treatment of asciminib in patients with CML, and market protection was granted for 10 years in the EU.

## Data Availability

No new data were generated or analyzed in support of this research.
